# The Association Between Borderline Personality Disorder Symptoms and Social Behaviour Among University Students

**DOI:** 10.3390/medicina61081465

**Published:** 2025-08-14

**Authors:** Andreea Sălcudean, Iustin Olariu, Mădălina-Gabriela Cincu, Ramona Amina Popovici, Iuliana Comșulea, Cristina-Raluca Bodo, Dora-Mihaela Cîmpian, Elena-Gabriela Strete

**Affiliations:** 1 Department of Ethics and Social Sciences, George Emil Palade University of Medicine, Pharmacy, Science and Technology of Târgu Mureș, 540142 Târgu Mureș, Romania; andreea.salcudean@umfst.ro (A.S.); cristina.bodo@umfst.ro (C.-R.B.); cimpiandora@umfst.ro (D.-M.C.); 2 Department of Dentistry, Faculty of Dentistry, “Vasile Goldiș” Western University of Arad, 310025 Arad, Romania; olariu.iustin@uvvg.ro; 3 Faculty of Medicine, George Emil Palade University of Medicine, Pharmacy, Science and Technology of Târgu Mures, 540142 Târgu Mureș, Romania; iulianacomsulea@yahoo.com; 4 Department of Management and Communication in Dental Medicine, Department I, Faculty of Dental Medicine, Victor Babes University of Medicine and Pharmacy of Timișoara, 300041 Timișoara, Romania; ramona.popovici@umft.ro; 5 Department of Psychiatry, George Emil Palade University of Medicine, Pharmacy, Science and Technology of Târgu Mureș, 540142 Târgu Mureș, Romania; elena.buicu@umfst.ro

**Keywords:** borderline, students, impulsivity, interpersonal difficulties, mental health

## Abstract

*Background and Objectives:* Borderline personality disorder (BPD) is a complex psychiatric condition characterized by emotional instability, impulsivity, a fluctuating self-image, and persistent difficulties in maintaining close interpersonal relationships. Among university students, these traits may be associated with social adjustment and academic functioning difficulties. The present study aimed to examine the prevalence of borderline traits within a Romanian student population and to investigate the associations between these traits and interpersonal difficulties encountered in family life, romantic relationships, and academic environments. *Materials and Methods:* This cross-sectional study included a total of 151 undergraduate students enrolled in higher education institutions across Romania. Data were gathered through an online questionnaire available between March and May 2025. The instrument comprised items addressing socio-demographic characteristics, diagnostic criteria for borderline personality traits according to the DSM, as well as self-reported social behaviour patterns. Statistical analysis was performed using GraphPad Prism 9, version 9.3.1 for Windows, employing Fisher’s exact test and the odds ratio (OR), with a significance threshold set at *p* < 0.05. *Results:* Most participants reported experiencing affective instability (71.5%) and distorted self-image (58.9%). Fear of abandonment was present in 29.4% of the respondents, while impulsivity was identified in 37.7%. Borderline personality traits were significantly associated with a range of social difficulties, including relational anxiety, outbursts of anger, peer conflicts, social withdrawal, and dissociative symptoms. Individuals who exhibited impulsivity, self-injurious behaviours, or dissociative episodes demonstrated a markedly increased risk of social dysfunction, with odds ratios ranging from 3 to 10 (*p* < 0.0001). *Conclusions:* The findings reveal a high prevalence of borderline traits within the analysed sample, along with statistically significant associations with social and emotional difficulties. These results underscore the importance of implementing psychological screening programs in universities, as well as early intervention strategies focused on the mental well-being of young adults. Establishing a supportive academic environment and fostering collaboration between faculty members and mental health professionals may play a key role in preventing symptom escalation and in promoting healthy personal and relational development.

## 1. Introduction

Borderline personality disorder (BPD) is a severe psychiatric condition characterized by a pervasive pattern of emotional, relational, and identity instability, often accompanied by impulsivity, self-destructive behaviours, and dissociative symptoms [1,2,3]. The estimated prevalence of BPD in the general population ranges from 1% to 6%, but this rate increases significantly among young individuals and those seeking mental health services [4,5,6]. Despite extensive international research on borderline personality traits in university students, there is a clear gap regarding studies conducted within the Romanian academic environment. Specifically, little is known about how these traits influence social and academic functioning among Romanian students. This study seeks to address this gap by providing a focused investigation of borderline traits and their relational impacts in Romanian higher education settings. Recent studies highlight university students as a population particularly vulnerable to the development of borderline traits, especially in the context of academic pressure, social transition, and the emotional instability typical of early adulthood [7,8,9]. The literature suggests that these traits are often associated with affective dysregulation, unstable relationships, self-harming behaviours, and challenges in social integration [10,11,12]. In addition, research indicates that childhood trauma, disorganized attachment, and maladaptive parenting styles represent major risk factors for the development of BPD [13,14,15]. International studies have shown that adolescents and young adults exposed to emotional abuse or severe neglect are more likely to develop borderline symptoms [16,17,18,19]. At a neurobiological level, BPD has been linked to dysregulation of the HPA axis, amygdala hypersensitivity, and reduced connectivity between the prefrontal cortex and limbic structures [20,21,22,23,24,25]. Studies conducted among university populations in both Asia and Europe have found consistent correlations between borderline traits and heightened anxiety, depressive symptoms, self-injurious behaviours, and relational difficulties [26,27,28]. However, in the Romanian context, there appears to be a lack of systematic investigation into these associations within academic settings, particularly regarding students’ social relationships and perceived support networks.

The primary objective of this research is to explore the presence of traits associated with borderline personality disorder among university students in Romania and to examine how these traits are linked to relational difficulties encountered in family, romantic, and academic contexts. Unlike previous studies that focus primarily on symptomatology or the validation of assessment tools, the present investigation seeks to highlight the deeper connections between the clinical and social dimensions of borderline personality. In addition, the study adopts an integrative approach that incorporates socio-demographic variables, the nine diagnostic criteria for BPD as defined in the DSM-5 [3], and items addressing relational dynamics and social behaviour. Through this multidimensional framework, this research aims to provide a comprehensive understanding of how borderline traits impact the social functioning of young adults.

According to the DSM-5 [3], borderline personality disorder is characterized by affective instability, impulsivity, distorted self-image, interpersonal dysfunction, and self-destructive behaviour. In non-clinical populations like university students, these traits often manifest as rapid mood changes, identity confusion, hypersensitivity to rejection, and difficulties in relationships. Affective instability involves intense mood swings triggered by interpersonal stressors, while distorted self-image reflects a fragile sense of self. Impulsivity refers to acting without considering consequences, often disrupting social or academic life. Fear of abandonment is an excessive worry about being rejected, and dissociative symptoms include emotional numbness or detachment in social situations.

Two main theories explain these features. Attachment theory suggests that inconsistent or disorganized caregiving in childhood leads to insecure relational patterns and heightened emotional reactivity in adulthood [14,15,16,17,18,19,20,21,22,23,24,25,26,27,28,29,30,31,32]. The biosocial model posits that borderline traits arise from the interaction between inherent emotional vulnerability and invalidating environmental responses, resulting in chronic emotional dysregulation and interpersonal difficulties [33]. University life’s pressures on identity consolidation, academic demands, and social expectations may exacerbate these vulnerabilities, increasing borderline symptomatology. Guided by these frameworks, this study investigates the prevalence of borderline traits among Romanian university students and their associations with social, familial, and romantic difficulties.

The originality of this study lies in its focus on a non-clinical, academic Romanian population, a group that has been largely understudied in previous BPD research. While the association between borderline traits and interpersonal difficulties is well established in clinical samples, this study expands current knowledge by examining how these traits are expressed in everyday social and academic contexts outside formal diagnosis. By operationalizing the DSM-5 criteria alongside self-reported social functioning items, the study offers a multidimensional perspective on the subclinical manifestations of BPD traits among university students.

The study is built around the following hypotheses:

**Hypothesis** **1.** *Students who experience fear of abandonment are more likely to encounter relational instability and difficulties in close social relationships.*

**Hypothesis** **2.** *Relational instability is associated with intense emotional reactions, challenges in social integration, and a weakened social support network.*

**Hypothesis** **3.** *Impulsivity and distorted self-image are associated with difficulties in emotional regulation and the emergence of dysfunctional behaviours in social and relational contexts.*

**Hypothesis** **4.** *Self-injury and dissociative symptoms are associated with high levels of emotional distress and difficulties in close social relationships.*

**Hypothesis** **5.** *Affective instability and feelings of inner emptiness are associated with social withdrawal, dissatisfaction with interpersonal relationships, and a disrupted sense of identity.*

Recent studies emphasize the importance of proactive screening within university settings, as well as the value of early psychological intervention, in order to prevent the progression of symptoms toward more severe clinical manifestations [29,30,31,32,33,34,35]. At the same time, there is evidence suggesting that social support networks play a protective role in promoting emotional stability and reducing self-destructive behaviours among individuals exhibiting borderline traits [34,35,36,37,38,39,40]. The present study offers a valuable contribution to the existing body of literature, with the potential to inform university-level mental health policies and guide targeted interventions for at-risk youth. At the same time, it provides a solid foundation for future longitudinal research and the development of prevention programs tailored to the specific needs of the Romanian academic environment.

The independent variables in this study are the borderline personality traits, conceptualized according to the DSM-5 diagnostic criteria, including emotional instability, impulsivity, fear of abandonment, and identity disturbance. The dependent variables are the social, emotional, and academic difficulties self-reported by university students, such as interpersonal conflicts, emotional dysregulation, social withdrawal, and problems with concentration or academic performance. These variables were analysed to examine the impact of borderline traits on students’ overall psychosocial adjustment.

## 2. Materials and Methods

**Figure 1:**
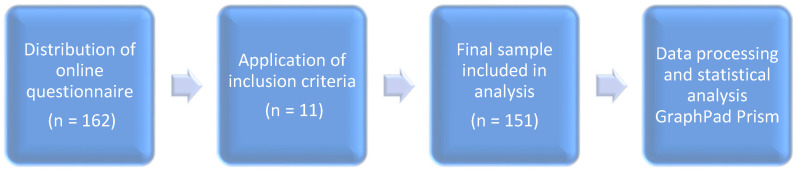
Flowchart illustrating the participant selection and data analysis process.

The research was conducted on a convenience sample of 151 students from various university centres across Romania. An overview of the process is presented in Figure 1. 

The inclusion criteria required participants to be at least 18 years of age and actively enrolled in university programs in Romania at the time of completing the questionnaire. The online survey was distributed across multiple Romanian university centres and was initially completed by 162 respondents. Following the application of eligibility criteria and the exclusion of incomplete or invalid responses (n = 11), the final sample included in the analysis consisted of 151 participants.

The questionnaire was distributed nationally via academic groups, student associations, and university social media platforms; the exact number and identity of participating institutions were not centrally documented. However, responses were received from students enrolled in a variety of academic disciplines and regions of the country, including urban centres known for their academic activity such as Târgu Mureș, Cluj-Napoca, Bucharest, Iași, and Timișoara. Given the voluntary and non-probabilistic nature of the sample, and the relatively small number of respondents (N = 151), this study was designed as an exploratory investigation. The primary objective was to identify potential associations between borderline personality traits and relational difficulties in a specific academic context, rather than to estimate prevalence in the general student population. The results should not be interpreted as representative of Romanian university students at large, but rather as a foundation for future research employing larger and stratified samples. Prior to participation, all individuals were informed about the purpose of the study and were asked to confirm whether they had ever been diagnosed with a psychiatric or personality disorder, or whether they were currently undergoing psychopharmacological treatment. Participants who reported such conditions were excluded from the final sample in order to reduce potential confounding effects and to maintain the focus on borderline traits in a non-clinical student population.

The study employed a cross-sectional design to investigate the relationships between borderline personality traits and students’ social and interpersonal difficulties. Participants completed an online questionnaire consisting of 30 items, organized into three sections: socio-demographic data (including gender, age, place of origin, field of study, year of study, and living situation), diagnostic criteria for borderline personality disorder as defined by the DSM-5, and items assessing the perception and frequency of relational difficulties in family, romantic, and peer contexts. Participants were selected using a non-probability, convenience sampling method based on voluntary participation. The online questionnaire was distributed through academic platforms, student networks, and social media channels, targeting students enrolled in various university centres across Romania.

The online questionnaire was distributed over a two-month period, between 17 March and 17 May 2025, with anonymity and voluntary participation fully ensured. The collected data were statistically analysed using GraphPad Prism version 9.3.1. for Windows. To identify associations between categorical variables, Fisher’s exact test was applied, and the strength of the relationships was assessed using the odds ratio (OR), with statistical significance set at *p* < 0.05. Fisher’s exact test was used due to the low frequencies in some cells, providing robust results for associations between binary categorical variables. Odds ratios were calculated to evaluate the strength of identified associations.

The study was reviewed and approved by the Research Ethics Committee of the “George Emil Palade” University of Medicine, Pharmacy, Science and Technology of Târgu Mureș, in accordance with Decision No. 3679 issued on 7 March 2025, confirming compliance with ethical standards for scientific research.

Given the recruitment strategy and the nature of the research instrument, the present study should be regarded as exploratory. The questionnaire used in this investigation comprised original items developed by the authors, grounded in DSM-5 diagnostic criteria and prior literature on borderline personality traits and interpersonal functioning.

The instrument consisted of three parts: sociodemographic data, a section assessing the presence of borderline traits through nine (yes/no) items, corresponding to the DSM-5 diagnostic criteria for borderline personality disorder, and a section evaluating social and academic difficulties through 15 items rated on a 4-point Likert scale (from “never” to “frequently”). These items aimed to capture behaviours such as impulsivity, emotional instability, relationship conflicts, social withdrawal, and concentration problems. The instrument has not undergone formal psychometric validation; it was constructed based on theoretical models and operational definitions to ensure conceptual clarity and construct relevance.

The questionnaire’s key constructs were clearly defined based on established criteria and the literature. For example, relational instability was measured by asking, “Do you feel that your close relationships are unstable, unpredictable, or frequently changing?” with “Sometimes” or “Frequently” indicating instability. Dissociative symptoms were assessed with “Have you ever felt as if you were outside of your body or emotionally disconnected from what was happening while around others?” Affective instability was evaluated by asking, “Do your emotions shift quickly and intensely, especially in reaction to interpersonal situations?” Impulsivity was identified through “Do you often act without thinking about the consequences?” Chronic emptiness was measured by the item “Do you often feel emotionally empty or numb, even when things are going well?” Social withdrawal included questions like “Do you avoid social contact or cancel plans with others because of your mood?” These clear operationalizations enhanced the validity and interpretability of the study’s results.

The instrument was not subjected to formal psychometric validation, and its reliability and construct validity were not evaluated. The findings should be interpreted as preliminary, and future research is encouraged to replicate and expand upon these results using standardized and validated assessment tools.

## 3. Results

### 3.1. Demographic Characteristics of the Sample of Students

Socio-demographic data included gender, origin, age, field and year of study, and living arrangements. Among 151 participants, most were female, from urban areas, aged 19–28 (mostly 21–25), living with others, and enrolled mainly in advanced years of medical programs. Participants were enrolled across all undergraduate years, with about 60% in their final years and 40% in their first or second year. This suggests that most had considerable academic experience. The study did not assess when borderline traits emerged; their higher presence in advanced students may indicate persistence or increased visibility with academic and social challenges.

### 3.2. Borderline Traits Identified According to DSM-5 Criteria

The second section of the questionnaire assessed the presence of borderline personality traits through nine items formulated in accordance with the diagnostic criteria outlined in the DSM-5. Participants responded using binary options (“yes” or “no”) based on their self-identification with each statement. The items covered key domains of the disorder: fear of abandonment, unstable interpersonal relationships, impulsivity, distorted self-image, affective instability, self-harming behaviours, intense anger, dissociative symptoms, and feelings of emptiness.

**Figure 2:**
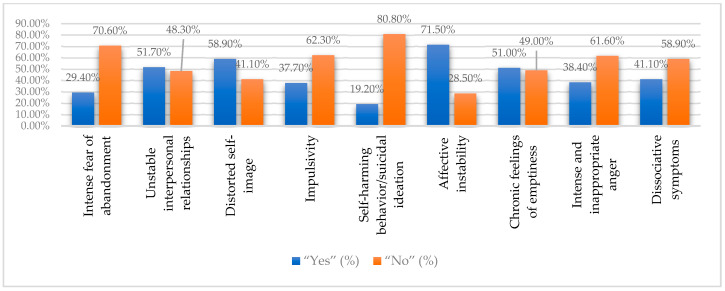
Prevalence of borderline personality disorder traits based on DSM-5 criteria (%).

Table 1 and Figure 2 show that affective instability (71.5%) and distorted self-image (58.9%) were the most common borderline traits among students, followed by feelings of emptiness (51%) and unstable relationships (51.7%). Impulsivity, dissociative symptoms, and intense anger were reported moderately, while self-harm was the least frequent (19.2%). These findings highlight the need for targeted psychological support addressing emotional and relational challenges in this population.

**Figure 3:**
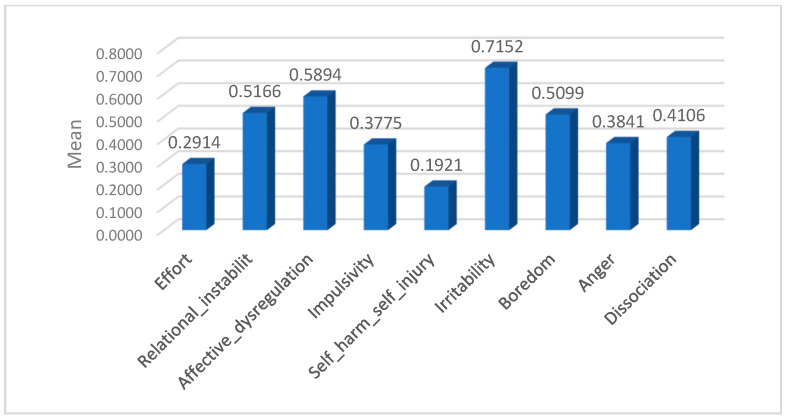
Mean scores representing the prevalence of borderline personality traits in the student sample.

Table 2 and Figure 3 present the descriptive statistics for the presence of specific borderline personality traits within the student sample (N = 151). Each trait was measured as a binary variable (0 = absence, 1 = presence), and the mean values indicate the proportion of participants endorsing each trait. The data reveal that irritability (mean = 0.715) and affective dysregulation (mean = 0.589) were the most commonly reported traits, followed by relational instability and feelings of boredom. Less frequently reported traits included self-harming behaviours and efforts to avoid abandonment. The standard deviations near 0.5 reflect considerable variability in the expression of these traits among the participants, suggesting a diverse profile of borderline features within this non-clinical university population.

These findings highlight a pronounced presence of emotional and interpersonal difficulties consistent with borderline personality traits in the sample. The prominence of affective instability and irritability underscores the central role of emotional dysregulation in this group. Self-harm and impulsivity were less prevalent; their presence in a significant subset of students points to the need for psychological awareness and targeted support. These results emphasize the importance of addressing emotional and relational challenges in university mental health initiatives.

The analysis of mean scores showed irritability as the highest reported trait (0.715), followed by affective dysregulation (0.589), relational instability (0.517), and boredom (0.510). Impulsivity (0.378), anger (0.384), and dissociation (0.411) had moderate prevalence, while self-harm was less common (0.192), and efforts to avoid abandonment were reported by 29% (0.291). These findings highlight significant emotional and interpersonal challenges in this student population.

Table 3 presents the descriptive statistics for the total borderline personality disorder (BPD) scores based on the sum of endorsed DSM-5 criteria among the 151 university students. The total scores ranged from 0 to 9, with a mean of 3.99 (SD = 2.63), reflecting a broad distribution of borderline trait severity within this non-clinical academic population.

These results indicate substantial variability in the overall expression of borderline personality traits, with some students exhibiting minimal symptoms and others showing moderate to high levels. This variability justifies the subsequent categorization of BPD levels into low and moderate to high groups to better understand the relationship between trait severity and social or academic difficulties.

The descriptive statistics highlight the prevalence and variability of borderline personality traits within the student sample. Notably, irritability and affective dysregulation were the most frequently reported traits, while self-harming behaviours appeared to be less common. These findings illustrate a diverse clinical profile within a non-clinical academic population. The moderate to high levels of BPD traits were defined as the endorsement of 5 or more DSM-5 criteria, distinguishing students with more pronounced borderline features from those with low trait presence (<5 criteria), as shown in Table 4. These descriptive statistics provide a solid foundation for subsequent analyses investigating the associations between borderline personality traits and social as well as academic difficulties in the student population.

**Figure 4:**
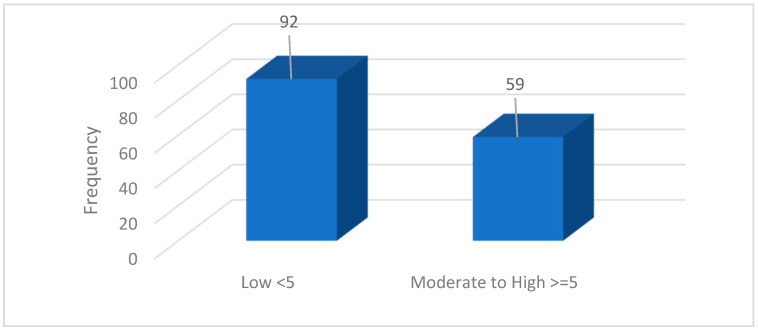
Frequency distribution of borderline personality disorder levels among university students.

The moderate to high BPD levels were defined as a total score of 5 or more DSM-5 criteria endorsed by the participants, while low levels correspond to fewer than 5 criteria. This cutoff allows differentiation between students with minimal versus more pronounced borderline traits (Figure 4).

The data indicate that a substantial proportion of students (39.1%) exhibit moderate to high levels of borderline personality disorder traits, which may predispose them to increased emotional and relational difficulties. This finding underscores the critical need for early identification and intervention strategies within university settings. Implementing psychological support programs tailored to students exhibiting elevated borderline traits can contribute to improving their academic success and overall mental well-being.

### 3.3. Perception of Social Networks and the Expression of Borderline Traits in the University Environment

The final section consisted of 15 items measuring how often participants experienced social and emotional difficulties related to borderline traits, including social withdrawal, trouble fitting in with peers, relationship conflicts, loss of interest in activities, and emotional regulation problems. Respondents chose from four frequency options (“Frequently” to “Never”), providing insight into dominant dysfunctional behaviours affecting both social and academic life. The key interpersonal difficulties reported are summarized in Table 5.

The most frequently reported difficulties (with ≥ 20 “Frequently” responses) were social withdrawal due to mood (n = 20), self-isolation from family (n = 29), perceived relational instability (n = 27), relationship-related anxiety (n = 21), anger outbursts in the family context (n = 20), and difficulty interacting with peers (n = 23). These findings highlight significant emotional and interpersonal challenges among students with borderline traits. While more severe behaviours such as threatening separation (n = 7) and social dissociation (n = 14) were less common, their presence in a non-clinical population suggests the need for targeted psychological support within academic settings.

## 4. Discussion 

To test the hypotheses formulated in this study, cross-tabulation analyses and Fisher’s exact test were employed, alongside the calculation of odds ratios (OR), in order to determine the presence of statistically significant associations between borderline personality traits and reported difficulties in social, academic, and relational domains. The significance threshold was set at *p* < 0.05.

**Hypothesis** **1.** *Students who experience fear of abandonment are more likely to encounter relational instability and difficulties in close social relationships.*

**Figure 5:**
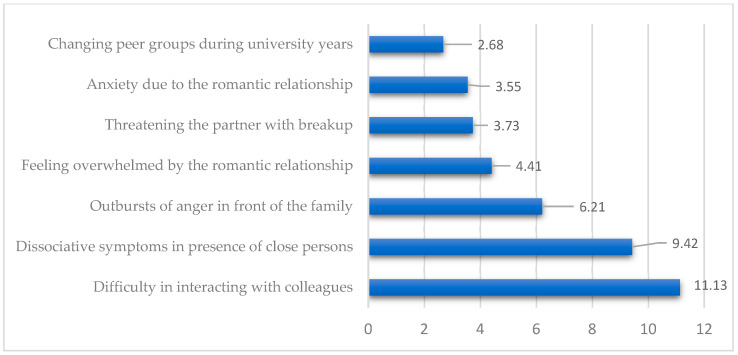
Odds ratios for significant associations between fear of abandonment and social behaviour in students.

To test Hypothesis 1, Fisher’s exact test and odds ratios were used. As shown in Table 6 and Figure 5, fear of abandonment was significantly linked to relational instability, social withdrawal, irritability toward friends, family conflicts, romantic anxiety, peer group changes, and dissociative symptoms (*p* < 0.05).

Figure 5 shows strong significant links between fear of abandonment and social difficulties, with the highest risks for trouble interacting with colleagues (OR = 11.13), dissociative symptoms (OR = 9.42), and anger outbursts at home (OR = 6.21). These results confirm that fear of abandonment greatly disrupts students’ social stability and relationships, supporting the first hypothesis.

**Hypothesis** **2.** *Relational instability is associated with intense emotional reactions, challenges in social integration, and a weakened social support network.*

To test Hypothesis 2, we analysed the associations between relational instability and various dysfunctional social behaviours using Fisher’s exact test and odds ratios. As shown in Table 7, relational instability was significantly linked to emotional outbursts, anxiety, social withdrawal, peer interaction challenges, frequent social circle changes, dissociative symptoms, and social dissatisfaction. These findings support the hypothesis.

Table 7 confirms Hypothesis 2, showing that relational instability is strongly linked to emotional and social dysfunctions. Students with unstable relationships had higher chances of anger outbursts, anxiety, social withdrawal, peer group changes, and difficulties interacting with colleagues. Notably, irritability toward friends (OR = 9.01), romantic anxiety (OR = 8.24), and dissociative symptoms (OR = 7.19) highlight the emotional strain and disrupted support typical of borderline relational patterns.

**Hypothesis** **3.** *Impulsivity and distorted self-image are associated with difficulties in emotional regulation and the emergence of dysfunctional behaviours in social and relational contexts.*

**Figure 6:**
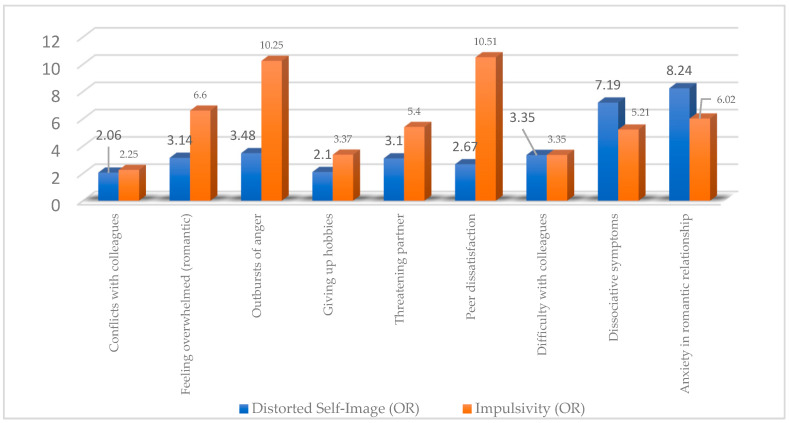
Comparative odds ratios for dysfunctional social and emotional behaviours associated with distorted self-image and impulsivity.

To test Hypothesis 3, Fisher’s exact test and odds ratios were used to analyse the data. As shown in Table 8 and Table 9 and Figure 6, impulsivity and distorted self-image were significantly linked to emotional outbursts, romantic anxiety, peer interaction difficulties, social dissatisfaction, threats of separation, dissociative symptoms, and social withdrawal.

The data presented in Table 8 support the hypothesis that a distorted self-image is associated with dysfunctional relational patterns and difficulties in emotional regulation. Students who reported this trait were significantly more likely to experience interpersonal conflicts, emotional outbursts, dissatisfaction in peer relationships, and threatening behaviour within romantic partnerships. Increased irritability, relational instability, and withdrawal from pleasurable activities such as hobbies reflect the negative impact of altered self-perception on students’ social and emotional functioning.

The data presented in Table 9 support the hypothesis that impulsivity is significantly associated with emotional dysregulation and dysfunctional social behaviours. Students who reported impulsive traits frequently exhibited emotional outbursts (OR = 10.25), anxiety within romantic relationships (OR = 6.02), and dissociative symptoms (OR = 5.21). They also experienced major difficulties in peer relationships, including dissatisfaction (OR = 10.51), a tendency toward social withdrawal, and threatening behaviours within intimate partnerships. These findings underscore the disruptive impact of impulsivity on students’ emotional and relational functioning.

Figure 6 supports Hypothesis 3 by showing that both distorted self-image and impulsivity are strongly linked to emotional dysregulation and maladaptive social behaviours. Impulsivity is associated with high odds of emotional outbursts, anxiety, and peer dissatisfaction, while distorted self-image relates more to dissociative symptoms and romantic anxiety. Together, these traits contribute to relational instability and poor emotional regulation in students with borderline tendencies.

**Hypothesis** **4.** *Self-injury and dissociative symptoms are associated with high levels of emotional distress and difficulties in close social relationships.*

To test Hypothesis 4, which posited that “self-injury and dissociative symptoms are associated with high levels of emotional distress and difficulties in close social relationships,” associations between these manifestations and a range of self-reported social and emotional behaviours were examined. Fisher’s exact test and the calculation of odds ratios (OR) were employed to identify statistically significant risks. The data presented in Table 10 reveal strong associations between self-aggressive or dissociative tendencies and outcomes such as social withdrawal, emotional outbursts, anxiety in relationships, difficulties interacting with close others, and withdrawal from enjoyable activities, thus confirming the proposed hypothesis.

The results presented in Table 10 confirm Hypothesis 4, demonstrating that both self-injury and dissociative symptoms are significantly associated with elevated levels of emotional distress and difficulties in close social relationships. Behaviours such as emotional outbursts, social withdrawal, anxiety in romantic relationships, dissatisfaction with peer connections, and disengagement from enjoyable activities were significantly more frequent among students who reported self-aggressive tendencies or dissociative experiences. This convergence supports the notion that these borderline traits exert a strong and direct impact on students’ socio-emotional functioning.

**Hypothesis** **5.** *Affective instability and feelings of inner emptiness are associated with social withdrawal, dissatisfaction with interpersonal relationships, and a disrupted sense of identity.*

To test Hypothesis 5, which proposed that affective instability and a persistent sense of inner emptiness are associated with social withdrawal, dissatisfaction with interpersonal relationships, and a disrupted sense of identity, the associations presented in Table 11 were analysed.

The data presented in Table 11 confirm Hypothesis 5. Both affective instability and the persistent sense of inner emptiness show significant associations with key indicators of social withdrawal (e.g., self-isolation from family, *p* = 0.0016, OR = 4.28 for affective instability; *p* = 0.0053, OR = 3.7 for inner emptiness), dissatisfaction with interpersonal relationships (e.g., peer conflicts, *p* = 0.0005, OR = 3.35 for inner emptiness; relational instability, *p* = 0.0095, OR = 3.24 for affective instability; *p* = 0.0001, OR = 6.49 for inner emptiness), and a distorted self-perception, reflected in dissociative symptoms (*p* = 0.012, OR = 2.55 for affective instability; *p* < 0.0001, OR = 4.61 for inner emptiness). These associations support a direct link between emotional dysfunction and difficulties related to social and identity integration among university students.

Our study found that 39.1% of Romanian university students show moderate to high levels of borderline personality traits (5 or more DSM-5 criteria). The most common traits were affective instability (71.5%), distorted self-image (58.9%), and relational instability (51.7%), linked to social and academic difficulties like anxiety, withdrawal, conflicts, and dissociative symptoms. Unlike Western samples with more externalizing behaviours, these students showed mainly internalizing symptoms, possibly due to cultural factors such as stigma and limited mental health services. The findings highlight the need for culturally sensitive screening and interventions in Romanian universities to support emotional regulation and social functioning.

The findings of this study confirm and expand upon previous evidence in the literature regarding the complex interplay between borderline personality disorder (BPD) traits and relational functioning within university populations. The high prevalence of emotional instability, distorted self-image, and interpersonal difficulties reflects the clinical profile of BPD, as documented in both clinical and non-clinical settings [5,6,26]. These results align with longitudinal research indicating that borderline traits often emerge and intensify during adolescence and early adulthood, a period marked by neurobiological vulnerability and heightened psychosocial demands [27,28]. Compared to studies from Western countries like the US, UK, and Scandinavia, which highlight overt self-harm, impulsivity, and substance use as key features of borderline personality traits among university students [26,28,40,41,42,43,44,45,46,47,48], our research reveals a predominance of internalizing symptoms such as social withdrawal, relational anxiety, and perceived instability in peer and romantic relationships. These differences may stem from cultural influences on emotional expression, stigma around mental health, and limited access to psychological services in Eastern Europe [7,47,49,50,51,52,53,54,55,56]. Our findings emphasize the need for culturally sensitive assessment and intervention approaches tailored to this context. By focusing on a largely understudied Eastern European non-clinical population, this study provides valuable empirical evidence that deepens the understanding of how sociocultural factors shape the manifestation of borderline personality traits.

Consistent with the hypotheses, core BPD features like fear of abandonment, impulsivity, and distorted self-image were strongly linked to social and emotional difficulties such as conflicts, withdrawal, irritability, dissociation, and dissatisfaction, supporting the role of affective dysregulation and attachment issues [29,30,31]. Impulsivity, related to decision-making deficits, correlated with dysfunctional relational patterns [32,33,34]. Standardized tools like the McLean Screening Instrument and the Zanarini Rating Scale are validated for university settings to aid early identification [35,36,37,38,39]. Pharmacological treatment is recommended mainly for comorbidities, while psychotherapy remains primary; however, overmedication without evidence-based therapy persists [40,41,42,43,44,45,46]. Effective therapies include DBT, Schema-Focused Therapy, and Mentalization-Based Therapy, though dropout rates challenge adherence [47,48,49,50,51,52,53]. The study also highlights inner emptiness, social integration problems, and distorted self-perception affecting academic and professional functioning [54,55,56,57,58,59,60,61]. The findings stress the need for university mental health policies with proactive, multidisciplinary services integrating screening, counselling, and educational support to prevent personality disorders and promote development. Recent research links emotional dysregulation to biological and contextual factors, such as gut microbiota, receptor expression, and family psychotherapy [62,63,64], as well as treatment adherence, COVID-19 impact, neuroinflammation, serotonin levels [65,66,67,68], and prenatal stress effects, emphasizing early prevention. Exposure to various forms of psychological or physical adversity has been associated with long-term emotional vulnerability, as highlighted in prior clinical assessments [69,70].

The findings of this study highlight important implications for university policies concerning mental health, early psychological intervention, and the development of institutional strategies to support students. The high prevalence of borderline traits, particularly affective instability, distorted self-image, and impulsivity, underscores the need for systematic psychological screening procedures within academic settings, even in the absence of a formal clinical diagnosis. It is recommended that universities implement integrated psychological services that include interventions focused on emotional regulation, identity development, and the enhancement of interpersonal skills. Psychoeducational workshops addressing relational anxiety, emotional instability, and social isolation may contribute significantly to reducing maladaptive behaviours and improving academic adjustment. Training academic staff to recognize early signs of psychological vulnerability is essential for facilitating timely access to specialized support. Interinstitutional collaboration between universities, clinical psychologists, and psychotherapists is strongly encouraged in order to build an inclusive educational climate that prioritizes mental health. Strategic investment in psychological support infrastructure should be regarded as a critical component of academic success and social integration for young people. Beyond the need for infrastructure development, the findings of this study carry meaningful clinical and social implications. The high prevalence of borderline traits, especially affective instability, impulsivity, and identity disturbances, suggests significant risks for students’ relational and academic functioning, even in the absence of a formal diagnosis. These traits may interfere with students’ ability to maintain stable relationships, regulate emotions, and remain engaged in academic life. Clinically, this calls for the implementation of targeted interventions aimed at emotional regulation, identity consolidation, and relational skills. Socially, these traits may increase the likelihood of isolation, academic withdrawal, and peer conflict, underscoring the importance of inclusive mental health strategies and proactive educational policies. Future longitudinal research may serve as a foundation for evidence-based public policy development, applicable within both national and international higher education systems.

Although this study provides valuable insight into the presence of borderline traits among Romanian university students and their impact on social and academic relationships, several methodological limitations must be acknowledged, as they may influence the interpretation of the results. First, the cross-sectional design of the research does not allow for causal inferences between personality traits and the reported relational difficulties. While statistically significant associations were identified, the directionality of the relationship between variables cannot be determined. Future studies should employ a longitudinal design to examine the development of these traits over time and to assess their impact on academic and social adjustment.

A key methodological limitation of this study is its reliance on a self-report questionnaire to assess borderline personality traits. Although the instrument was aligned with DSM-5 criteria and prior literature, it is important to acknowledge that personality disorders, particularly BPD, are often associated with limited insight, which may compromise the accuracy of self-assessment.

Structured clinical interviews, such as the SCID-5-PD, are considered the gold standard for diagnostic evaluation. However, given the exploratory scope and non-clinical nature of this study, such methods were not feasible. As a result, the findings should be interpreted as indicative of borderline traits rather than as diagnostic of BPD. Future studies should incorporate validated clinician-administered instruments to enhance diagnostic precision.

Another limitation of this study concerns the methodological status of the research instrument. Although the questionnaire was conceptually grounded in DSM-5 criteria and existing theoretical models, it has not been psychometrically validated, and no tests of internal consistency or factorial structure were performed. The use of a non-probabilistic convenience sample based on voluntary participation may introduce selection bias. The relatively small sample size (N = 151) limits the statistical power and prevents generalizations regarding the actual prevalence of BPD traits in the broader Romanian student population.

Individuals with a heightened interest in mental health or those already experiencing emotional difficulties may have been more inclined to respond. The gender imbalance, with a predominance of female participants, also limits the generalizability of the findings to the wider student population. These factors reduce the representativeness and diagnostic precision of the results. The study offers relevant exploratory insights and may serve as a foundation for future large-scale investigations employing validated instruments and stratified sampling methods.

This study focused on borderline traits and relational difficulties but did not assess comorbidities like depression, anxiety, or PTSD, which often affect symptom expression and social functioning in BPD. Future research should use larger, more diverse samples and longitudinal, mixed-methods designs to track symptom progression and explore protective factors such as social support and emotional regulation. Evaluating psychiatric comorbidities will help understand their role in student vulnerability. These directions can improve tailored interventions for Romanian students. The study found a high prevalence of borderline traits, especially affective instability, distorted self-image, and emptiness, linked to social integration issues, conflicts, irritability, withdrawal, and dissociation, indicating increased psychosocial risk.

## 5. Conclusions

The present study revealed a concerningly high prevalence of borderline traits among Romanian university students, particularly regarding affective instability, distorted self-image, unstable interpersonal relationships, and feelings of inner emptiness. The findings confirmed the proposed hypotheses, demonstrating that these traits are significantly associated with social difficulties, including anxiety in close relationships, frequent interpersonal conflicts, social withdrawal behaviours, and dissociative symptoms. The statistical analyses indicate an elevated risk of emotional and relational dysfunction among young individuals exhibiting these traits, in line with international literature highlighting the vulnerability of university populations to personality disorders.

The study underscores the urgent need to integrate educational and clinical policies that recognize the importance of mental health within academic environments. The implementation of regular psychological screening programs is essential for the early identification of at-risk individuals, along with the development of accessible psychological support services tailored to the psychosocial profile of students. Evidence-based psychotherapeutic interventions conducted in collaboration with academic staff may help reduce symptom severity and improve students’ social, academic, and professional functioning. The results provide a valuable framework for reflecting on how emotional and relational difficulties interfere with the educational process and with personal identity development during the transition to adulthood. Student mental health must be regarded as a fundamental component of academic success and social cohesion. This study makes an important contribution to the existing literature by offering empirical foundations for future research and for the design of targeted, feasible, and sustainable interventions within both Romanian and international university settings. The study’s findings hold important clinical and social implications. The strong association between borderline traits and dysfunctional relational patterns calls for the implementation of targeted psychological interventions within university settings, even in the absence of formal diagnoses. Culturally sensitive programs focused on emotional regulation, interpersonal skills, and identity development are essential for supporting at-risk students. By highlighting internalizing manifestations such as social withdrawal and relational anxiety rather than externalizing behaviours more frequently reported in Western samples, this research contributes to a nuanced understanding of borderline personality features within Eastern European academic populations. These insights offer valuable directions for both national mental health policy and future cross-cultural investigations.

## Figures and Tables

**Table 1:** Frequency of “Yes” and “No” responses for each DSM-5 diagnostic criterion of borderline personality disorder.

Evaluated Criterion	“Yes” (n)	“No” (n)
Intense fear of abandonment	44	107
Unstable interpersonal relationships	**78**	73
Distorted self-image	**89**	62
Impulsivity	57	94
Self-harming behaviour/suicidal ideation	29	122
Affective instability	**108**	43
Chronic feelings of emptiness	**77**	74
Intense and inappropriate anger	58	93
Dissociative symptoms	62	89

Note: Criteria highlighted in bold represent the most frequently reported traits (n ≥ 75).

**Table 2:** Descriptive statistics for the presence of borderline personality traits in the student sample.

Descriptive Statistics						
	N	Min.	Max.	Mean	Std. Error	Std. Deviation
Effort	151	0.00	1.00	0.2914	0.03710	0.45592
Relational_instability	151	0.00	1.00	**0.5166**	0.04080	0.50139
Affective_dysregulation	151	0.00	1.00	**0.5894**	0.04017	0.49358
Impulsivity	151	0.00	1.00	0.3775	0.03958	0.48637
Self_harm_self_injury	151	0.00	1.00	0.1921	0.03216	0.39523
Irritability	151	0.00	1.00	**0.7152**	0.03685	0.45281
Boredom	151	0.00	1.00	**0.5099**	0.04082	0.50156
Anger	151	0.00	1.00	0.3841	0.03971	0.48800
Dissociation	151	0.00	1.00	0.4106	0.04017	0.49358

Note: Values highlighted in bold represent the borderline personality traits most frequently reported by participants.

**Table 3:** Descriptive statistics of total borderline personality disorder scores in the student sample.

Descriptive Statistics						
	N	Minimum	Maximum	Mean	Std. Error	Std. Deviation
BPD_total	151	0	9	**3.9868**	0.21387	2.62802

Note: Important values are highlighted in bold.

**Table 4:** Distribution of borderline personality disorder levels among university students.

BPD_level			
	Frequency	Percent	Cumulative Percent
Low < 5	92	60.9	60.9
Moderate to High ≥ 5	**59**	**39.1**	100
Total	151	100	

Note: Important values are highlighted in bold.

**Table 5:** Frequency of reported difficulties according to each evaluated dimension.

Evaluated Construct/Thematic Label	Never (n)	Rarely (n)	Sometimes (n)	Frequently (n)
Familial relationship distress	23	53	55	**20**
Conflicts with peers	60	67	19	5
Romantic relationship distress	61	35	40	15
Anger outbursts in family context	45	58	28	**20**
Social withdrawal due to mood	19	70	42	**20**
Loss of interest in hobbies	53	42	38	18
Threatening separation in romantic relationships	101	25	18	7
Irritability toward friends	16	59	61	15
Self-isolation from family	27	46	49	**29**
Perceived relational instability	28	47	49	**27**
Relationship-related anxiety	50	40	40	**21**
Dissatisfaction in peer relationships	28	56	56	11
Difficulty interacting with peers	23	60	46	**23**
Changing social circles during university	48	61	32	10
Social dissociation (feeling “outside the body”)	70	40	27	14

Note: Constructs highlighted in bold represent the most frequently reported difficulties (“Frequently” ≥ 20 responses).

**Table 6:** Associations between the effort to avoid abandonment and social behaviour among students.

Social Behaviour	*p*-Value	OR (95% CI)
Feeling overwhelmed by the relationship with family	0.21	-
Conflicts with colleagues	0.06	-
Feeling overwhelmed by the romantic relationship	**0.0004**	**4.41 (1.93–9.18)**
Outbursts of anger in front of the family	**0.0003**	**6.21 (2.17–17.03)**
Cancelling meetings with close ones due to mood	0.06	-
Giving up hobbies due to boredom	0.7	-
Threatening the partner with breakup	**0.0005**	**3.73 (1.73–7.82)**
Irritability toward friends	**0.0034**	**-**
Social withdrawal from family	**<0.0001**	**-**
Relational instability	**<0.0001**	**-**
Anxiety due to the romantic relationship	**0.0042**	**3.55 (1.47–9.22)**
Dissatisfaction with peer relationships	**<0.0001**	**-**
Difficulty in interacting with colleagues	**0.0027**	**11.13**
Changing peer groups during university years	**0.02**	**2.68 (1.16–6.03)**
Dissociative symptoms in presence of close persons	**<0.0001**	**9.42**

Note: Bolded values indicate statistically significant associations.

**Table 7:** Associations between relational instability and social behaviour.

Social Behaviour	*p*-Value	OR (95% CI)
Feeling overwhelmed by the relationship with family	**<0.0001**	-
Conflicts with colleagues	0.06	-
Feeling overwhelmed by the romantic relationship	**<0.0001**	**7.15**
Outbursts of anger in front of the family	**<0.0001**	**4.53 (2.08–9.92)**
Cancelling meetings with close ones due to mood	**0.02**	**3.46 (1.12–9.07)**
Giving up hobbies due to boredom	0.73	-
Threatening the partner with breakup	**<0.0001**	**5.63 (2.57–11.85)**
Irritability toward friends	**0.001**	**9.01**
Social withdrawal from family	**0.0002**	**6.29**
Relational instability (autoreferential)	**<0.0001**	**-**
Anxiety due to the romantic relationship	**<0.0001**	**8.24**
Dissatisfaction with peer relationships	**0.0001**	**6.71**
Difficulty in interacting with colleagues	**0.0116**	**3.64 (1.32–9.63)**
Changing peer groups during university	**0.0018**	**3.08 (1.55–6.11)**
Dissociative symptoms in the presence of close persons	**<0.0001**	**7.19**

Note: Bolded values indicate statistically significant associations.

**Table 8:** Associations between distorted self-image and social behaviour.

Social Behaviour	*p*-Value	OR (95% CI)
Feeling overwhelmed by the relationship with family	0.11	-
Conflicts with colleagues	**0.04**	**2.06 (1.04–3.95)**
Feeling overwhelmed by the romantic relationship	**0.0013**	**3.14 (1.56–6.23)**
Outbursts of anger in front of the family	**0.001**	**3.48 (1.67–7.24)**
Cancelling meetings with close ones due to mood	0.62	**-**
Giving up hobbies due to boredom	**0.03**	**2.10 (1.09–4.15)**
Threatening the partner with breakup	**0.0029**	**3.10 (1.46–6.65)**
Irritability toward friends	**0.0058**	**5.1**
Social withdrawal from family	0.12	-
Relational instability	**0.0097**	**3.23 (1.42–7.62)**
Anxiety due to the romantic relationship	0.07	-
Dissatisfaction with peer relationships	**0.03**	**2.67 (1.19–5.97)**
Difficulty in interacting with colleagues	0.25	-
Changing peer groups during university	0.85	-
Dissociative symptoms in the presence of close persons	0.13	-

Note: Bolded values indicate statistically significant associations.

**Table 9:** Associations between impulsivity and social relationships.

Social Behaviour	*p*-Value	OR (95% CI)
Feeling overwhelmed by the relationship with family		**7.91**
Conflicts with colleagues	**0.0263**	**2.25 (1.14–4.54)**
Feeling overwhelmed by the romantic relationship	**<0.0001**	**6.6**
Outbursts of anger in front of the family	**<0.0001**	**10.25**
Cancelling meetings with close ones due to mood	**0.010.0018**	**6.07**
Giving up hobbies due to boredom	**0.0016**	**3.37 (1.55–7.22)**
Threatening the partner with breakup	**<0.0001**	**5.4**
Irritability toward friends	**0.0005**	-
Social withdrawal from family	**<0.0001**	-
Relational instability	**<0.0001**	-
Anxiety due to the romantic relationship	**<0.0001**	**6.02**
Dissatisfaction with peer relationships	**0.0002**	**10.51**
Difficulty in interacting with colleagues	**0.03**	**3.35 (1.16–9.48)**
Changing peer groups during university	0.07	-
Dissociative symptoms in the presence of close persons	**<0.0001**	**5.21 (2.51–10.70)**

Note: Bolded values indicate statistically significant associations.

**Table 10:** Comparative associations of self-harm and dissociative symptoms with dysfunctional emotional and social behaviours.

Social Behaviour	*p*-Value (SH)	OR (SH)	*p*-Value (Diss)	OR (Diss)
Outbursts of anger	**0.001**	**6.45**	**<0.0001**	**4.9**
Social withdrawal	**0.0003**	**5.87**	**0.0001**	**6.78**
Anxiety in romantic relationship	**0.007**	**3.75**	**0.002**	**4.61**
Peer dissatisfaction	**0.005**	**4.33**	**0.003**	**3.95**
Giving up hobbies	**0.02**	**2.65**	**0.01**	**2.88**

Note: Bold values indicate statistically significant results at *p* < 0.05.

**Table 11:** Associations between affective instability/feelings of emptiness and social behaviours.

Social Behaviour	Affective Instability (*p*)	Affective Instability (OR)	Emptiness (*p*)	Emptiness (OR)
Feeling overwhelmed by family relationship	0.22	-	0.26	-
Conflicts with colleagues	0.85	-	**0.0005**	**3.35**
Feeling overwhelmed by relationship with romantic partner	**0.0061**	**2.77**	0.09	-
Outbursts of anger towards family	0.23	-	**0.0006**	**3.75**
Cancelling plans with close ones due to mood	**0.005**	**4.29**	**0.027**	**3.36**
Giving up hobbies due to boredom	0.85	-	**<0.0001**	**7.87**
Threatening partner with breakup	0.12	-	0.06	-
Irritability towards friends	0.39	-	0.11	-
Social withdrawal from family	**0.0016**	**4.28**	**0.0053**	**3.7**
Perception of relational instability	**0.0095**	**3.24**	**0.0001**	**6.49**
Anxiety due to romantic relationship	**<0.0001**	**4.61**	0.29	-
Dissatisfaction with peer relationships	**0.0095**	**3.24**	**0.0007**	**5**
Difficulty interacting with colleagues	0.12	-	**0.0414**	**2.75**
Changing friend groups during university period	0.43	-	0.29	-
Dissociative symptoms in the presence of close others	**0.012**	**2.55**	**<0.0001**	**4.61**

Note: Values highlighted in bold indicate statistically significant associations (*p* < 0.05).

## Data Availability

The data used and/or analysed during the current study are available from the corresponding author upon reasonable request. Unfortunately, these data are not publicly available because of privacy or ethical restrictions.

## Abbreviations

The following abbreviations are used in this manuscript:

BPD	Borderline Personality Disorder
DSM-5	Diagnostic and Statistical Manual of Mental Disorders, Fifth Edition
OR	Odds Ratio
DBT	Dialectical Behaviour Therapy
PTSD	Post-Traumatic Stress Disorder
